# Not the same CURE: Student experiences in course-based undergraduate research experiences vary by graduate teaching assistant

**DOI:** 10.1371/journal.pone.0275313

**Published:** 2022-09-27

**Authors:** Emma C. Goodwin, Jessica R. Cary, Erin E. Shortlidge

**Affiliations:** Department of Biology, Portland State University, Portland, Oregon, United States of America; City University of New York, UNITED STATES

## Abstract

To expose all undergraduate science students to the benefits of participating in research, many universities are integrating course-based undergraduate research experiences (CUREs) into their introductory biology laboratory curriculum. At large institutions, the bulk of introductory labs are instructed by graduate teaching assistants (GTAs). Graduate students, who are often teachers and researchers in training, may vary in their capacity to effectively teach undergraduates via the CURE model. To explore variation in GTA teaching and the subsequent outcomes for students, we used a case study research design at one institution where introductory biology students participate in GTA-taught CURE lab sections. We used multiple data sources, including in-class focus groups, worksheets, and surveys to explore student perceptions of the GTA-led CURE. Students perceived variation both in the ability of their GTAs to create a supportive and comfortable learning environment, and in the instructional priorities of their GTAs. We also compared student and GTA perspectives of student engagement with research elements in the CURE. While GTAs were divided in their perceptions of whether the CURE provided students with the opportunity to experience the element of relevant discovery, most students—regardless of their GTA—did not perceive that relevant discovery was emphasized in the CURE. Finally, individual GTAs seemed to influence how students perceived why they were participating in the CURE. These data imply that students in CUREs may have vastly different and potentially inequitable research experiences depending on their instructor.

## Introduction

Participation in apprentice-based research can be a transformative experience for undergraduates in Science, Technology, Engineering, and Math (STEM) fields. Research experiences can result in a wide range of outcomes including increased interest in science, motivation to pursue a STEM career, and ultimately retention in STEM [1–6]. However, opportunities for undergraduate research are inherently constrained by inadequate space and limited resources in faculty-led labs, thus access to research is often inequitable [7]. STEM departments and universities are expected to address the issue by ensuring that all STEM undergraduates have a chance to participate in a research experience [8–11].

One approach to increasing undergraduate participation in research is to integrate research-based courses into STEM curricula, especially at the introductory level where students seem to differentially benefit from the broad positive impacts associated with research participation [7, 9, 12]. A common model for integrating research into the curriculum is via course-based undergraduate research experiences (CUREs). In CUREs, students generally participate in research projects under the guidance of an instructor, within the structure of a standard-enrollment laboratory course [13]. A commonly-used framework from the CURE literature outlines that undergraduates should specifically engage in five elements inherent to research: 1) use of multiple scientific tools and practices (*Scientific Practices*); 2) *Collaboration* both with other students and advanced scientists, who may be the course instructors; 3) *Iteration*, such that students have opportunities to revise their experiments and understand how scientific research builds off of previous research; 4) potential for *Novel Discovery* (i.e., experiments that address questions where the answer is unknown within the broader scientific community); and 5) *Broader Relevance*, such that the research problem is relevant and meaningful to other scientists or a local community who are not involved in the CURE [13]. Throughout this paper, we refer to these five research elements as the “CURE constructs.”

Like apprentice-based research, participation in CUREs results in benefits for students, such as increased scientific skills and understanding of the process of science, increased scientific self-efficacy, interest, and motivation in science, and increased retention in STEM [14–19]. Although occurring in the classroom setting, CUREs are meant to provide students with a “real research experience,” and students who have participated in multiple types of CUREs have reported that they indeed believe they are participating in real research [15, 20, 21]. We have begun to explore which elements of CUREs lead students to believe that the research experience is real, and have found that experiencing failure and challenges in the process of conducting science may be a key contributor to students’ beliefs that they are participating in real science [20]. Further, the perception that a CURE can hold the potential for novel and relevant research discoveries is linked to positive student experiences in a CURE, specifically, students’ sense of project ownership and motivation to engage in the CURE [21, 22]. However, we do not know the extent to which individual instructor behaviors can impact student experiences in CUREs.

The CURE format differs from traditional “cookbook” labs in many ways, including an increased focus on student independence because students must grapple with the often ‘unpredictable’ nature of conducting real science [23]. Such curricular differences necessitate instructional approaches that may differ from those used to teach cookbook labs. Faculty instructors of CUREs have identified CURE-specific skillsets and traits that are important for successful CURE instruction: 1) the ability to deal with the uncertainty of research; 2) a background in scientific research and specific proficiency in the area of research that is the focus of the CURE; and 3) a willingness to invest the necessary time and effort [24].

Many of the studies demonstrating benefits for students participating in CUREs were conducted in classrooms taught by PhD-level faculty instructors. These studies neglect to consider an important logistical consideration of large-scale implementation of CUREs—at 91% of research institutions graduate teaching assistants (GTAs), rather than faculty, provide the bulk of the laboratory instruction [25]. Students have different perceptions of faculty and GTA instructors of laboratory courses: students report that faculty instructors in general tend to be more enthusiastic, organized and prepared, and to have greater knowledge than GTAs [26]. In contrast, students perceive GTA instructors to be less confident, yet create a more relaxed and laid-back environment [26]. Therefore, employing GTAs rather than faculty instructors could impact how students perceive and benefit from a CURE.

Evidence suggests that GTAs, who are often novice teachers and researchers, may struggle in their capacity to successfully teach a CURE [27, 28]. GTAs do not always embrace their role as a research mentor in a CURE classroom, and some perceive high emotional and logistical costs with teaching the CURE, including feeling unprepared to serve as research mentors, feeling they lack appropriate expertise, and struggling with the time commitment required to teach a CURE [27, 28]. While some GTAs may have the expertise and motivation to capably teach a CURE, others may lack these attributes, which could negatively impact the experiences of students in their classes.

To date, little work has directly explored the impacts of individual instructors on CURE outcomes. Some studies have reported preliminary evidence that students are indeed impacted by variability in the quality of their instruction—for example, one study found that students of a single PhD-level CURE instructor had a much lower proportion of students who could “think like a scientist” at the end of the term, compared to students of other instructors in the study [14]. Another study of nearly 800 students who enrolled in multiple CURE sections of the same course over a two-year period saw statistically significant variation in content knowledge gains for students across CURE sections, and the researchers suggested this variation could be attributed to the 30 different GTAs involved in teaching the CURE sections [18]. Further, a recent study explicitly focused on the individual pedagogical behaviors of GTA instructors of CUREs, and the impact their behavior has on student outcomes: Esparza and colleagues [29] compared the pedagogical actions of four GTA instructors of CUREs with four GTA instructors of traditional laboratory courses, and found that GTAs in the CURE overall tended to engage more frequently in interactive classroom behaviors such as posing questions or one-on-one student interactions. However, there was significant variation in the instructional behaviors among the four CURE GTAs, and regardless of course type, GTAs who engaged more frequently in interactive instructor behaviors positively impacted students’ autonomous motivation, self-efficacy, and collaboration [29]. All three of these studies demonstrate that the behaviors of individual CURE instructors are likely variable and result in differential outcomes for students, but we are left without a clear understanding of the potential implications of instructor affect and actions on student experiences in a CURE.

Instructor behavior is likely to impact students’ buy-in, or attitudes and willingness to engage in a CURE [30–32]. Our previous work has considered student buy-in and beliefs regarding the authenticity of a research experience offered through an independent CURE curriculum [20], but the extent to which instructors impact student buy-in has not been explored. Student buy-in to engage within active-learning contexts is influenced by their beliefs that active learning is beneficial to their education [30]; we therefore hypothesized that student buy-in to engage in the CURE will be impacted by their beliefs regarding their institution’s intentions in employing CUREs in the introductory biology curriculum. We expect that beliefs about why CUREs are being integrated into the curriculum are likely influenced by the information conveyed to students via their instructors, as well as the behaviors of the instructors themselves.

Here we study the alignment of how individual GTAs perceive and instruct a CURE and how their students experience and perceive that CURE. Specifically, we address three research questions:

1. *How do undergraduate perceptions of their learning environment and the instructional priorities in a CURE classroom differ by GTA*?
2. *How do student and GTA perceptions compare regarding the research elements scaffolded in a CURE*?
3. *Why do students think their university has them engage in a CURE in introductory biology*, *and does this vary by GTA*?

We addressed these questions through a case study research design, comparing the experiences of students in multiple lab courses taught by different GTAs within a single large-enrollment introductory biology course. This case study approach allowed us to focus on the impacts of individual GTA instructors without introducing confounding contextual variables (e.g., different institutions, CURE design). We used multiple data sources, collected from both students and their GTAs (outlined in Table 1), to allow for a deep and multi-faceted understanding of the experiences and perspectives of our study participants.

**Table 1:** Data collection summary.

Data Source	Participants	Administration	Associated Research Questions ^a^
In-Class Modified Focus Groups	n = 406 (students, 20 focus groups)	Late-term, in-person, during class	1
Lab Priorities Worksheet	n = 376 (students)	Late-term, in-person, during class	1 & 2
Demographics and Laboratory Course Assessment Survey	n = 383 (students) n = 9 (GTA instructors)	End-of-term, online survey platform	2
GTA Interviews	n = 9 (GTA instructors)	Late-term, in-person	2
Student Reflection Questions	n = 351 (students)	Mid-term, online survey platform	3

^a^ Study Research Questions: 1) How do undergraduate perceptions of their learning environment and the instructional priorities in a CURE classroom differ by GTA? 2) How do student and GTA perceptions compare regarding the research elements scaffolded in a CURE? 3) Why do students think their university has them engage in a CURE in introductory biology, and does this vary by GTA?

## Methods

### Preliminary work

In spring 2019, we conducted a pilot study in preparation for the study described throughout this manuscript. Our pilot study was conducted at a comprehensive university in the Western US with CUREs embedded throughout the undergraduate laboratory science curriculum. We collected data (including interviews, focus groups, course observations, and surveys) from eight GTAs teaching CURE labs and 119 of their students. While these data are not included in this manuscript, we used the pilot data to inform the design of this current study, including the crafting of reflection and interview questions, as described below.

### Study context

In fall 2019, we conducted an extensive study within a large-enrollment introductory biology laboratory course at a research-intensive university in the Pacific Northwest, where students co-enrolled in an introductory biology lecture course and a weekly CURE lab section. We used a multiple-case study design, where each “case” encompassed the experiences and perceptions of a single GTA and that GTA’s students [33].

We studied the 20 lab sections associated with the introductory biology course, each with approximately 22 students per section. All lab sections were taught by one of nine GTAs, seven of whom had taught the same lab course at least once previously. See Table 2 for the total number of sections and students taught by each GTA and GTAs’ prior teaching experience with the course. Though the CURE lab sections were a co-requisite with a larger lecture course, content-wise, they were largely independent of the lecture course with minimal curricular overlap or discussion about the CURE during the lecture portion. A “network CURE” was implemented in the lab sections, where students participated in the HHMI SEA-PHAGES curriculum [34]. Over the past decade, the SEA-PHAGES curriculum has been widely implemented at many different institutions worldwide, with varying course structures [34, 35]. This network curriculum has resulted in many publications, both in education research [e.g., 36, 37] and basic science [e.g., 38, 39].

**Table 2:** Number of sections taught and student study participants for each GTA.

GTA Identifier (n = 9)	# of Sections Taught (n = 20)	# of Student Participants per GTA (n = 434)^a^	# of terms GTA had taught CURE^b^
A	2	45	1
B	2	39	5
C	3	69	2
D	3	64	3
E	2	47	2
F	2	44	1
G	2	35	4
H	2	46	2
J	2	45	5

^a^ Total number of participants summed from each GTA’s combined sections at the start of the term, which does not account for enrollment attrition throughout term.

^b^ Total number of terms that the GTA had taught the SEA-PHAGES CURE as a graduate student, inclusive of the current term.

In the SEA-PHAGES curriculum, students experience the five CURE research constructs [13] in that: they collaborate in groups (*Collaboration*) on a single term-long research project, where they collect soil samples and attempt to isolate and characterize bacteriophages (*Scientific Practices*). Students have the opportunity to repeat experimental steps when needed, both in-class and during several open-lab hours offered each week (*Iteration*). Due to the extensive diversity of soil bacteriophages, successfully isolated bacteriophages are presumed to be previously undescribed by scientists (*Novel Discovery*), and students then catalog their phages in a national database where the information has potential to be scientifically useful in the future (*Broader Relevance*). At our study institution, students worked in teams of three or four to isolate and characterize a single bacteriophage, and within a single GTA’s lab section there were generally five or six teams each concurrently working on their own team’s bacteriophage.

GTAs in our study received some training prior to teaching the CURE: each GTA, regardless of whether they had already taught the curriculum, was required to attend a two-day “boot camp” at the beginning of each term. In this boot camp, they were reminded of the purpose of engaging students in a CURE and discussed the data that support the impact of CUREs on students. New GTAs additionally received practical training at the beginning of the term to learn relevant lab techniques. Finally, all GTAs attend a required meeting each Friday throughout the term, where they received additional training as needed and engaged in collaborative discussions to support their CURE instruction. Additional instructional support, advice, assistance, and mentorship related to their CURE instruction was provided by the faculty lead instructor and a full-time lab coordinator.

### Ethical approval and participant consent

This study was approved by the Portland State University Institutional Review Board (no. 196388–18). All study participants (GTAs and undergraduate students) were consenting adults. We obtained written consent from undergraduate student participants for all the data collection points described below, including the in-class focus groups, lab worksheet, end-of-term survey, and student reflection questions. We also obtained written consent from GTAs for their participation in interviews and the end-of-term survey.

### Data collection: In-class modified focus groups

To explore how students perceived the CURE, two researchers led modified focus groups within each lab section during Week 14 of the 16-week term. We based our modified focus group design on that of the “Small Group Analysis” protocol [40, 41]. The focus groups were conducted with students during class time, without the presence of the course instructors. The researchers first introduced themselves to the students, explained why they were there, handed out consent forms, and explained that students would earn course points if they participated in the focus group and associated surveys (described below). Students had the option to complete an alternative assignment to earn the course credit if they chose not to participate in the study. All students (100%) in attendance chose to participate in the study (n = 406 students, 20 lab sections). Students sat in their regular groups of 2–4 students per table, and the focus groups were audio and video recorded.

Informed by findings from our pilot study, we iteratively designed three question prompts to facilitate discussion for the focus groups. We conducted face-validity checks with several undergraduates outside of our final study population to confirm that questions were clear and interpreted as intended [42]. We posed the following three questions to students:

1. Please describe specific things that your [lab] instructor did that supported your learning and overall experience in this lab course.
2. Please describe what your lab classroom environment feels like (i.e., the mood, or general attitude of your lab mates and instructors). What made you feel this way?
3. Please describe any specific things your instructors could have done that would have improved your experience during this lab course.

The researchers stated the first prompt (above) aloud and gave students two minutes to reflect and write down their own thoughts. Students then had about three minutes to discuss their responses to the prompt within their small groups. The researchers then facilitated a full-class discussion on the prompt, asking for volunteers from each group to share their thoughts. Researchers invited other students in the class to respond by elaborating, disagreeing, or confirming what their classmates were saying. This process was repeated for each of the three prompts.

### Data collection: Lab learning priorities worksheet

Immediately after the class discussion described above, students were asked to individually complete a worksheet, which was designed to probe student perceptions of what aspects of the course they thought were particularly important to their GTA. We intentionally had students respond to the worksheets after the focus group discussions because we have previously found that providing subjects with reflective activities prior to responding to interview questions can elicit thoughtful responses [27].We therefore wanted students to have spent some time thinking about their experiences in class (during the discussions) prior to responding to the worksheet in order to allow students to better recall their experiences as they filled out the worksheets. We wanted to reduce the likelihood that students rush through the worksheets with minimal reflection. We recognize that the class discussions may have impacted students’ responses, but the two activities were designed to address differing aspects of the lab environment.

To explore student perceptions of what was most important to their GTA, we developed a list of 15 hypothetical learning priorities for the CURE, encompassing different experiences that we expected students may find relevant to their CURE. This list of learning priorities was partially informed by our analysis of pilot data collected from students participating in a CURE, where students answered open-ended survey questions and interview questions that probed their perceptions of the purpose of participating in the CURE and their understanding of what their instructors wanted them to get out of the CURE. Additionally, we drew from literature about hypothesized outcomes of CURE participation [43] and the defined CURE constructs [13] when developing and refining the list of lab learning priorities. The worksheet instructions and full item list are included as Supporting Information (S1 Text). The lab priorities worksheet asked students to indicate what they projected to be the three most and three least important lab learning objectives to their GTA in the CURE lab. Students were also asked to specify what they believed was the single most and single least important learning objective to their GTA and provide a written rationale for each. Because we developed the learning priorities list based both on published literature and preliminary data on student perceptions of the purpose of a CURE, some items were more or less relevant for this particular CURE: for example, the item, “Students better understand the content of the associated lecture course”, was not particularly expected to rank highly, whereas we expected that students may rank “Students learn the importance of revising or repeating their work to improve the quality of their research” more highly.

### Data collection: End-of-term laboratory course assessment survey

We administered an end-of-term survey to GTAs and students across sections via *Qualtrics* to quantitatively assess GTA and student perceptions of research elements in the CURE and to collect demographic information. This survey included the Laboratory Course Assessment Survey (LCAS), a 17-item instrument designed to measure CURE students’ perceived participation in the CURE constructs of *Collaboration*, *Broader Relevance/Novel Discovery*, and *Iteration* [44]. Because the LCAS is designed to measure several of the original CURE constructs, we hypothesize that higher scores on the LCAS would be indicative of student perceptions of a more “CURE-like” experiences. We modified the frequency-related response options to better suit the weekly course format, as described by Goodwin and colleagues (2021; for modified item list see Table A in S2 Text). GTAs completed a modified version of the student LCAS, with identical items and response options as in the student version but with framing modified to reflect the GTA’s instructional role [45]. For example, an item on the student survey that read “I was encouraged to reflect on what I was learning” became “I encouraged my students to reflect on what they were learning” on the GTA version of the survey. Like the other instruments used in this study, the instructor-modified LCAS was first used in the pilot study for this work.

### Data collection: GTA interviews

GTAs teaching each of the CURE lab sections were recruited to participate in end-of term interviews. Interviews were conducted in-person and were audio-recorded. A subset of interview questions was developed to explore GTA perceptions of the presence of the five CURE constructs in their lab sections, as we were interested in how their responses would align with their students’ perceptions of experiencing the CURE constructs. Further description of the interviews and analysis are described in Goodwin et al. (2021) [27].

### Data collection: Student reflection questions

During the eighth week of the term, we administered a constructed-response survey to students via the online survey platform *Qualtrics*. This survey included the question “Why do you think your institution wants students to participate in the research-based curriculum offered in this lab?”

### Data analysis: In-class modified focus groups

To analyze focus group data, two researchers watched the recordings of the modified focus groups together and individually made detailed notes and descriptions of the ideas that emerged during whole-class discussions. This resulted in a preliminary list of codes, or short phrases that described a reoccurring idea in the discussions. We then reviewed the preliminary codes together and organized them into the broad categories of “GTA Strengths” and “GTA Weaknesses.” Preliminary codes that did not clearly represent strengths or weaknesses of the GTA were coded into additional categories, but these codes were not used for further analysis. The GTA “Strengths” and “Weaknesses” categories included themes that grouped together specific codes: for example, “GTA Strengths” included the theme “GTA is a strong communicator.” Within the “strong communicator” theme were codes such as “GTA explained experimental purpose well” and “GTA communicates at appropriate level for students.” The researchers then re-watched the recordings together, and using the final codebook, coded every focus group to consensus by discussing throughout the recording which codes were most appropriate.

We realized that many of the codes, independent of the theme in which they were grouped, were indicative of distinct competency levels regarding the GTA’s management of the CURE and ability to support a positive learning environment. We therefore developed four additional categorizes to re-organize the codes as they related to a GTA’s competency: “Above and beyond,” “Baseline,” “Insufficient,” and “Help!”. To determine which of the codes aligned with each of these four categories, two researchers individually considered each focus group code and made an independent judgment about the category in which each code belonged. The researchers then compared their individual code-category alignment decisions and discussed to resolve any initial disagreements. The final GTA competency categories are described below:

1. ***Above and beyond***. Codes in this category indicated that the GTA supports student learning and creates a positive environment for learning. Examples include “GTA used inclusive/effective teaching techniques” and “GTA was invested in students and teaching.”
2. ***Baseline***. Codes in this category indicated that the GTA completes the basic tasks of managing a CURE. Examples include “GTA communicated expectations well” and “Most students understand the purpose of the CURE research project.”
3. ***Insufficient***. Codes in this category described minor critiques of the GTA that detracted from student learning. Examples include “Needed more instructional clarity or guidance for lab procedures” and “More organization needed from GTA.”
4. ***Help*!** Codes in this theme described major critiques of the GTA in their capacity to support student learning. Examples include “Lack of engagement from GTA” and “GTA creates a stressful environment.”

A list of the final themes and codes, as well as the GTA competency categorization of each code, is included as supporting information (S1 Table).

Each GTA taught two or three lab sections, and after our initial coding of each focus group, we compared coded segments for sections taught by the same GTAs. Qualitatively, we did not perceive notable differences between the individual focus groups of students with the same GTA, which aligned with our perceptions while conducting the focus groups. We therefore decided to continue our analyses of the focus groups by GTA, rather than by class section. We summed the total code count by GTA for each of the above competency categories. To normalize code counts between GTAs who taught different numbers of sections, and to account for the fact that certain focus groups may have simply been more talkative than others, we then divided the total code count for each competency category by the total code count of all the competency-related codes that arose in each of the GTA’s sections. This produced the relative frequencies of each competency category mentioned by students of each GTA.

### Data analysis: Lab learning priorities worksheet

Completed worksheets were screened by researchers, and worksheets were excluded from analysis if they a) were not filled out correctly, or b) their response in the open-ended question implied they did not interpret the question as intended. Out of the 406 students who completed the worksheet, 376 responses were deemed usable. We calculated the percent of students who said that a given objective was among the top three most or least prioritized by their GTA overall, and additionally disaggregated this information by GTA. This allowed us to identify similarities and differences in student perceptions in how their GTA prioritized each lab learning objective.

### Data analysis: End-of-term laboratory course assessment survey

Although there is evidence that the LCAS has produced valid data at other institutions with undergraduate students in CUREs (the population for which the LCAS was designed), different student populations may interpret survey items uniquely [46]. We therefore used confirmatory factor analysis (CFA) to test if the latent construct structure of the instrument functions as expected in our student population [47]. We tested a correlated three-factor model with the CURE constructs *Collaboration*, *Broader Relevance/Novel Discovery*, and *Iteration* as three separate latent factors, using a robust maximum likelihood estimator with the Satorra-Bentler correction to correct for potential non-normality in our item responses. After evaluating reliability and data-model fit statistics (described further in S2 Text), we averaged the item responses for each construct. We found that individual student construct scores generally mirrored each other (i.e., students of a given GTA generally scored high or low on the *Collaboration* construct and scored similarly high or low on the *Broader Relevance/Novel Discovery* and *Iteration* constructs). We therefore decided to simplify our analyses by summing each student’s average construct score to create a single LCAS metric approximating the degree to which students perceived essential CURE elements. We conducted an ANOVA to assess if there were differences in LCAS scores of students taught by different GTAs and used Tukey’s HSD post-hoc tests to further explore potential differences. All statistical analyses described throughout this manuscript were conducted in R version 4.0.5, using the base, *lavaan* and *psych* packages [48–51].

The instructor-modified version of the LCAS was administered to all nine GTAs. Due to the small sample size, we did not conduct a CFA to assess how the LCAS functions among our GTA population. As for the student factor scores, we averaged item responses within each construct for each GTA, and summed the average construct scores.

### Data analysis: GTA interviews

To analyze interviews, we developed an initial provisional codebook informed by the CURE literature, our pilot study and previous work with GTA and faculty instructors of CUREs. Two researchers read all GTA interview transcripts and generated additional codes or clarified *a priori* codes as needed to best capture GTA ideas expressed during interviews. Part of this codebook was specifically designed to analyze GTA perceptions as it related to the presence or absence of each of the CURE constructs (for example, “GTA perceived Relevant Discovery in CURE;” “GTA perceived lack of Relevant Discovery in CURE”). Both researchers used the final codebook to independently code each interview, and then reviewed and discussed each code designation to consensus. Finally, a single researcher re-read through each interview to check that coding was accurate and consistent across interviews.

### Data analysis: Student reflection questions

We conducted initial open coding [52] on students’ written responses to the question “Why do you think your institution wants students to participate in the research-based curriculum offered in this lab?”, and two researchers developed and refined the initial list of codes while iteratively reading approximately 20% of the student responses. Researchers then individually coded all student reflection responses, including the 20% used in codebook development. Throughout the coding process, researchers met regularly to reconcile their individual coding decisions, and all final coding designations were discussed by both researchers to consensus. After coding was complete, we organized the codes in response to this question into two major themes: 1) codes indicating that a student believed that their university implements CUREs for student-centered purposes, and 2) codes indicating that a student believed that their university implements CUREs for non-student-centered purposes. We used Kruskall-Wallis tests to assess whether there were differences among GTAs in the proportion of their students who expressed these perceptions and conducted post-hoc tests (Dunn’s test with the Benjamini-Hochberg adjustment) to further explore potential differences.

## Results

### Participant information

Demographic information was collected from the end-of-term survey, which 383 undergraduate students and all nine GTAs completed. The majority (70%) of undergraduate students self-identified as women, and the average age was 19.8 years old (SD = 1.9 years). Most students were sophomores (56%), and over 90% of students reported no prior research experience. About 20% of students were pursuing a biology degree, and an additional 75% were pursuing other STEM degrees.

Of the nine GTAs, six were pursuing PhDs and three were pursuing Master’s degrees. While none of the GTAs were studying bacteriophages or virology (which would be most relevant to the SEA-PHAGES CURE), eight were pursing biology degrees and one was pursuing a STEM degree outside of biology. Six of the GTAs identified as women; six identified as white, and the average age of GTAs was 29.5 years old (SD = 5.2 years). GTAs completed their undergraduate degrees at a wide array of institution types, including doctoral, Master’s-granting, primarily undergraduate institutions, and international institutions. Six of the nine GTAs had teaching experience outside of the CURE, which included experience as a teaching assistant for other courses, experience teaching at international institutions, and experience teaching at the K-12 level.

### GTAs vary in their capacity to create a supportive classroom environment

**Fig 1:**
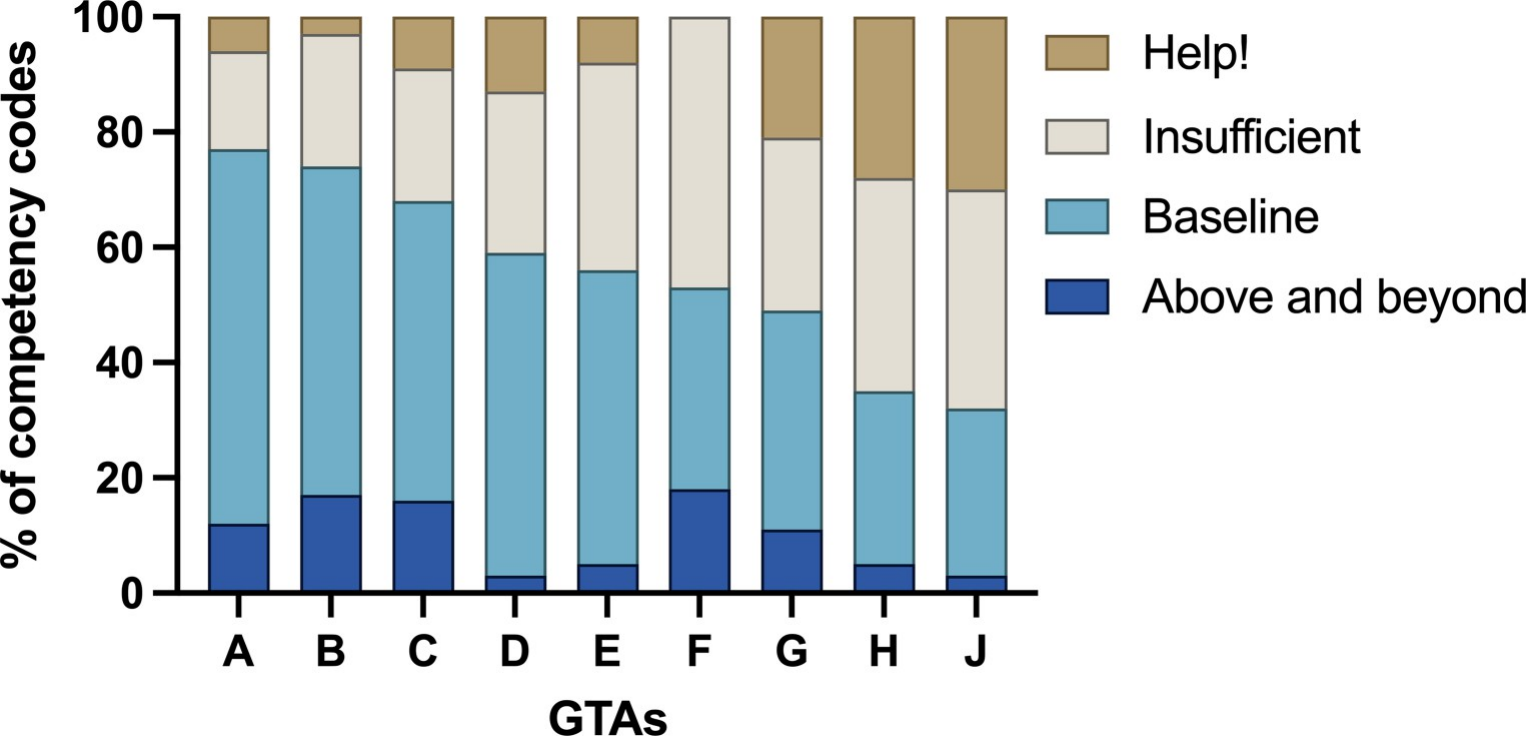
GTAs varied in their ability to create a positive learning environment. Student descriptions of their GTA’s competency during focus groups were coded to compare the frequency at which students describe their GTA’s actions as highly competent (“Above and beyond”), meeting expectations (“Baseline”), not meeting expectations (“Insufficient”) or highly incompetent or destructive of the classroom environment (“Help!”). GTAs are ordered from A to J by decreasing proportions of positive (i.e., “Above and beyond” and “Baseline”) codes. More than half of the statements made by students of GTAs A through F represented these positive environment codes, while more than half of the statements made by students G through J represented adverse (“Insufficient” or “Help”) codes.

We partially address our first research question (“How do undergraduate perceptions of their learning environment and the instructional priorities in a CURE classroom differ by GTA?”) through the modified in-class focus group data, which allows us to explore differences in how students perceive their CURE classroom environment. Within the focus groups, students of six GTAs (GTAs A through F) generally felt that their GTA was competent in promoting a positive classroom environment (“Above and beyond,” or “Baseline” codes, Fig 1). Illustrative quotes below were sourced from the modified focus groups.

Students who discussed that their GTA was highly competent in creating a positive classroom environment (“Above and beyond” codes) described an appreciation for the “extra” effort their GTA put into the class, that their GTA clearly demonstrated investment in their learning, and that the lab experience was not just productive but also enjoyable. During the focus groups, one student explained how their GTA was particularly understanding of student needs and willing to put extra effort in to accommodate their students:

*“Our TA is helpful*, *especially if you need help with something and can’t make it to a lab, or a make-up lab. They’re willing to help you out because they understand that we’re all busy. Everybody’s busy and helping each other out makes everyone’s life a little bit easier.”–A student of GTA B*

While students occasionally described instances where they perceived their GTA’s competency in the CURE was “Above and beyond” their expectations, throughout our focus groups students more frequently described positive attributes of their GTAs that we categorized as “Baseline.” These codes described instances when GTAs appeared to be meeting what might be expected of one teaching any course, such as clearly communicating expectations and the procedures and purpose of the course, providing thorough feedback to students, and fostering a comfortable, productive, and collaborative environment. Students valued the effort their GTAs put into making sure these baseline needs were met in their lab class:

*“Our TA would always go over the protocol no matter what*, *so that was reassuring if you didn’t quite understand it before coming to lab. I really liked that they would send a weekly email telling us what we could expect in lab and what assignments were due. There’s a lot of things going on, so it was nice to have that.”—A student of GTA E*

Students of all GTAs described instances where their GTA was “Insufficient” in meeting student needs—though students of some GTAs described considerably more instances of this than others (Fig 1). Students who found their GTAs to be “Insufficient” described needing their GTA to be more organized, have clearer expectations, make better use of class time, and that their GTA lacked some understanding of the course material. A common observation made by students during the focus groups was that their GTA did not provide enough information or context to allow them to fully understand the purpose of what they were doing in the CURE:

*“It would help if [our GTA] gave an explanation for which substances did what in the experiments*, *because I often found myself thinking: ‘Oh, the instructions say [to add this] so I might as well add it’ without understanding the purpose for adding it… I have no idea why we have to add this substance.”–A student of GTA J*

These perceived “Insufficient” instances were often frustrating for students, but overall were minor and ultimately did not prevent students from succeeding in the CURE or feeling comfortable in the classroom. However, students also described more alarming instances where their GTA failed to provide sufficient support, presenting challenges to students having a positive and beneficial experience in the CURE. We coded these instances as “Help!” (Fig 1). In these instances, students often described feeling that the classroom environment was uncomfortable or stressful:

*“[Our GTA] gets really frustrated with us sometimes when we don’t understand*. *We can ask them a question and then they’ll try to explain it to us, but they’ll just say stuff we really don’t understand and then [our GTA] gets really frustrated with us. We feel how frustrated they’re getting and there are definitely moments where I’m like: Are you going to shake me? Like ‘Understand!’”—A student of GTA G*

While most students ultimately spent more time discussing positive aspects of their GTA-taught CURE, students of three GTAs (G, H, and J) described more “Help!” and “Insufficient” instances than “Baseline” or “Above and beyond” (Fig 1) The experiences of students taught by these the GTAs appear to be very different from the experiences of their peers, in that they ultimately do not seem to experience sufficient support from their GTA or feel like their classroom is a comfortable learning environment.

### GTAs emphasize different learning priorities in a CURE

**Fig 2:**
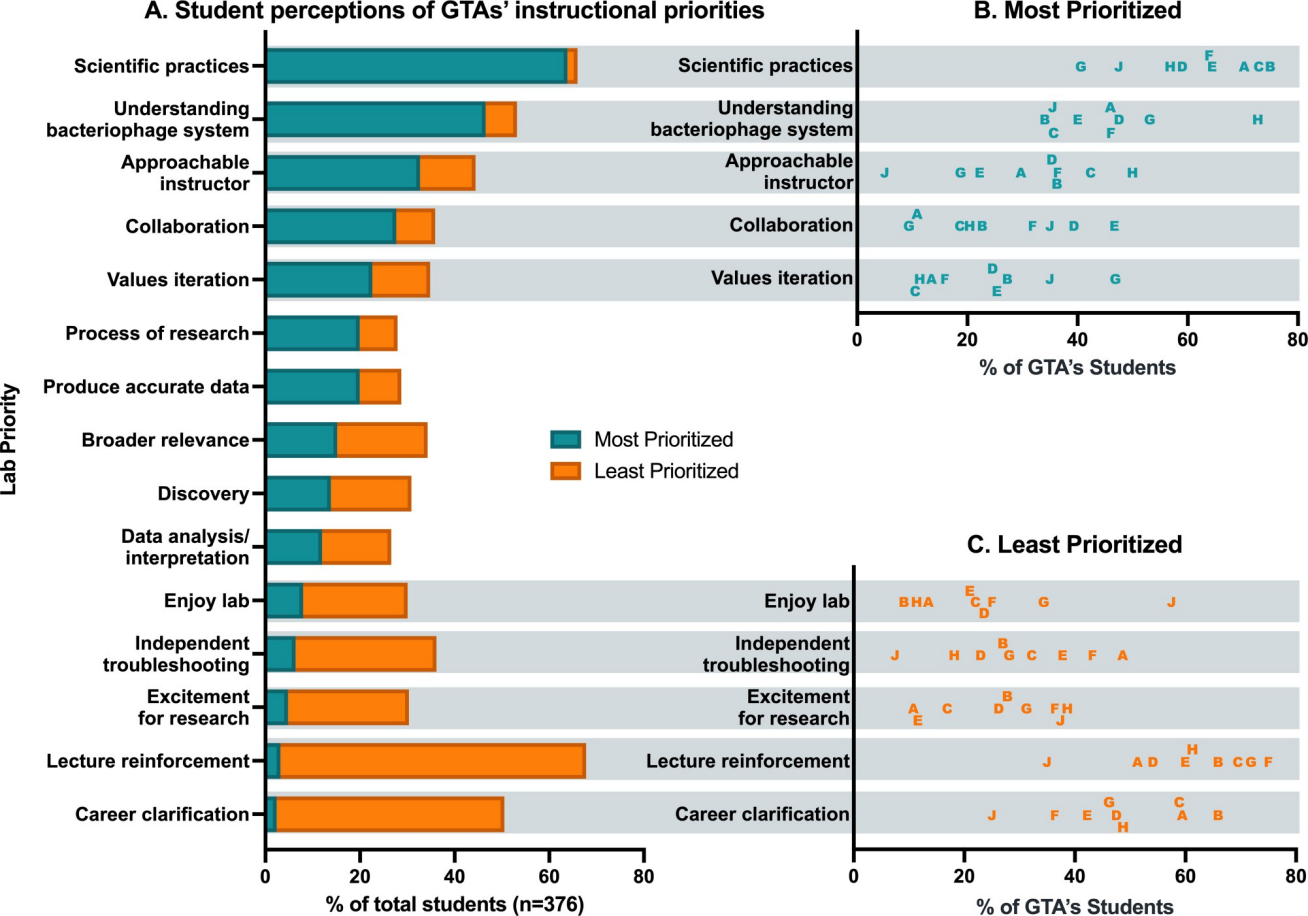
GTAs emphasized different lab priorities. (A) The percent of students overall who reported that each item was among the most (blue bar) or least (orange bar) prioritized for their GTA. Students reported that items near the top of the panel (i.e., "Scientific practices"; “Understanding bacteriophage system”) were most prioritized by their GTA, while items near the bottom of the panel (i.e., “Lecture reinforcement”; “Career clarification”) were least prioritized. (B) The percent of each GTA’s students who reported that specific items were *most* prioritized by their GTA. (C) The percent of each GTA’s students who reported that specific items were *least* prioritized by their GTA. Letters in (B) and (C) identify the students of individual GTAs, demonstrating that students of different GTAs differed in their perspectives of the importance of each item to their GTAs.

We continued to explore our first research question (“How do undergraduate perceptions of their learning environment and the instructional priorities in a CURE classroom differ by GTA?”) with the student lab priorities worksheets, where students indicated which aspects of the course they perceived to be most and least prioritized by their GTA. This allowed us to explore how a GTA’s differential interests or priorities, *as perceived by students*, may lead students to experience the CURE differently. Overall, given the options we provided, students perceived that developing basic lab skills, developing an understanding of the bacteriophage and host system, and being comfortable approaching the instructor with questions were highly prioritized by their GTAs (Fig 2A). In contrast, students reported that better understanding the content of the lecture portion of the course, learning if they are interested in a research career, and learning to troubleshoot problems independently were the least-emphasized course aspects (Fig 2A).

Perceptions of how GTAs prioritized certain items did not vary considerably by GTA. For example, few students, regardless of GTA, reported that experiencing the “process of research,” “producing accurate data,” experiencing “broader relevance” and “discovery” in the course, and learning “data analysis/interpretation” were high priorities for their GTA. However, students’ perceptions how other items were prioritized varied widely by GTA (Fig 2B and 2C). For example, nearly 50% of GTA E’s students believed that “collaboration” was one of the highest priorities of their GTA, while approximately 10% of GTA A and G’s students listed collaboration as a priority (Fig 2B). This implies that GTA E is likely emphasizing collaboration—a critical CURE element—more than GTAs A and G, and students of different GTAs are therefore experiencing the CURE differently. Other areas of high variation between students’ perceived experiences in a CURE are highlighted by the items included in Fig 2B, where 50% of GTA H’s students said that being an “approachable instructor” was important to their GTA. On their lab priorities worksheet, one student explained how GTA H emphasized their GTA’s approachability, explaining:

*“I was confused a lot and [the GTA] always made the room feel comfortable to ask questions and be open talking with them*.”

In contrast, only 5% of GTA J’s students said that instructor approachability was important in their class, and many of GTA J’s students emphasized in their worksheets that this was particularly unimportant:

*“A lot of students are scared to ask questions from fear of getting [the GTA] mad and making us feel as if we know nothing*.”

Students also perceived variance in the items that were least prioritized by their GTAs (Fig 2C). For example, nearly 60% of GTA J’s students reported that having students “enjoy” the lab was among the least prioritized by their GTA, while on average only 20% of students taught by other GTAs indicated this was specifically a low priority for their GTA.

### Students and their GTAs disagree on the presence of the CURE constructs in their class

We used student and GTA responses to the LCAS, student perspectives from the lab priorities worksheets, and GTA interviews to address our second research question, where we compare student and GTA perceptions of the implementation of the CURE research constructs (*Scientific Practices*, *Collaboration*, *Iteration*, *Novel Discovery/Broader Relevance*).

**Fig 3:**
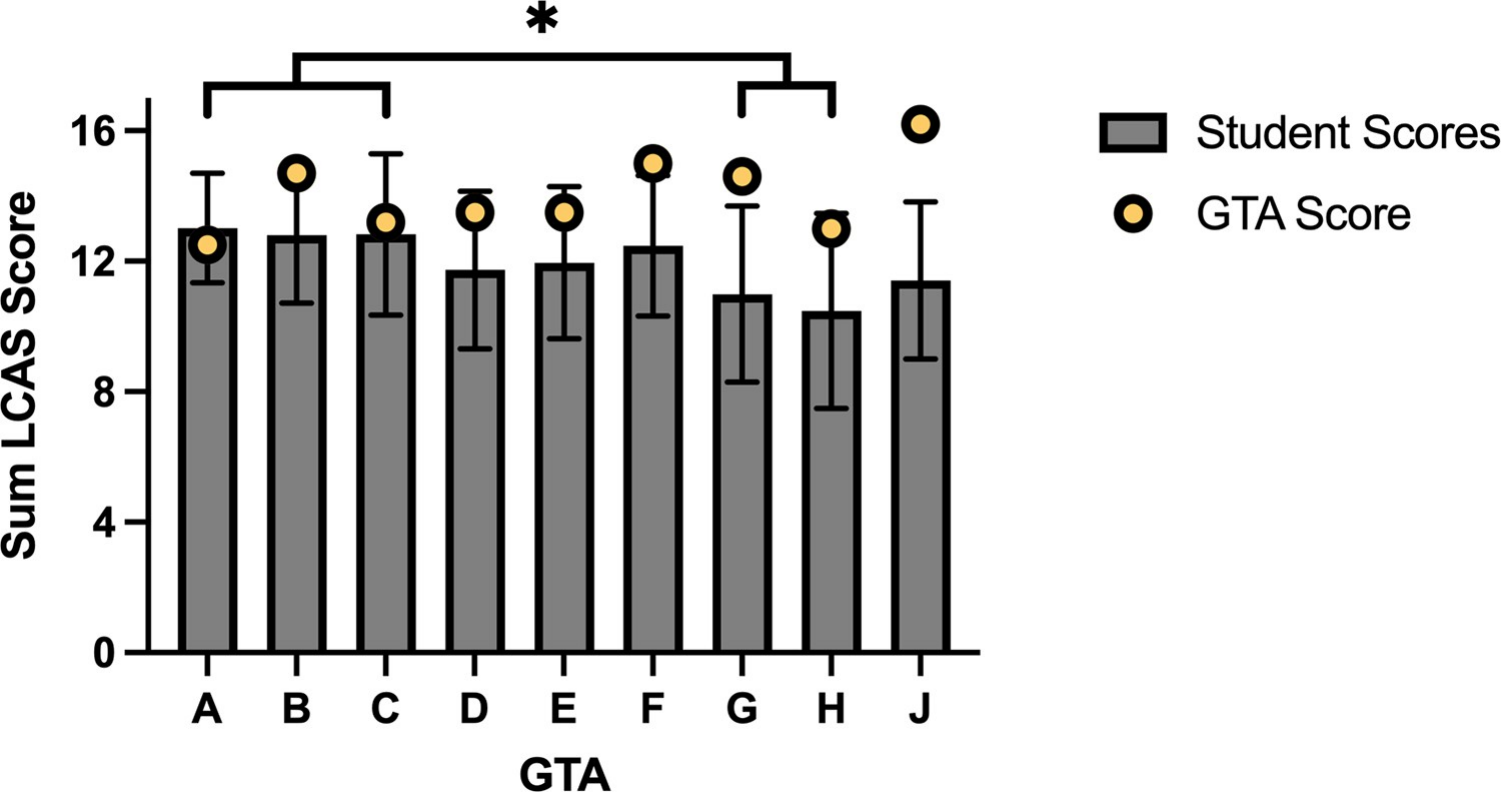
GTAs impact student perceptions of essential CURE constructs, and perceive greater presence of CURE constructs in the curriculum than their students. Grey bars represent the summed average (± 1 SD) of the three constructs measured in the student LCAS (*Collaboration*, *Iteration*, and *Broader Relevance/Novel Discovery*) for students of each GTA. Students of GTAs A, B, and C perceive significantly higher elements of research in their classes than do students of GTAs G and H. Yellow circles represent the summed average of the three constructs for each GTA’s LCAS score, allowing for comparison of the GTA’s perspective of facilitating Collaboration, Iteration, and Broader Relevance/Novel Discovery to student’s perceptions of experiencing those research elements. Most GTAs perceive similar or slightly higher levels of research elements in their CURE classrooms as their students. However, three GTAs (F, G, and J) perceive notably higher levels of these research elements in their class than their students.

Descriptive statistics for each LCAS survey item in the student version of the survey revealed that no items deviated significantly from normal (Table B in S2 Text), and we used a robust estimator in the CFA to account for moderate deviations from normality. Though all three subscales had acceptable internal consistencies, fit indices for the final model fall at or slightly short of commonly used guidelines for “acceptable” model fit (for further discussion, see S2 Text), and thus our student survey data should be considered with caution. That said, we did find that students of GTAs A, B, and C scored significantly higher on the LCAS than students of GTAs G and H (Fig 3), implying that students in classes taught by GTAs A, B, and C perceive experiencing higher levels of research.

We compared students’ responses to the LCAS to their GTA’s response on the instructor version of the LCAS, which measured GTA perceptions of facilitating the CURE constructs. GTA LCAS scores represent the score of a single instructor at a single point in time, and due to our low GTA sample size we do not have the ability to assess how the GTA version of the LCAS functions in our study population, as we did for the student LCAS data. However, we found that GTA scores were often within one standard deviation of their student’s LCAS scores, though tended to land higher than their student’s mean score. LCAS scores for GTAs F, G, and J were more than one standard deviation higher than their student’s LCAS scores, indicating that these GTAs overestimated the degree to which they facilitated CURE constructs compared to their student’s perceptions of experiencing the CURE constructs.

Data from the lab priorities worksheets provided additional insight to student perceptions of individual CURE constructs as fostered by their GTA: on average, 61% (SD = 11.5%) of each GTA’s students believe that learning *Scientific Practices* is one of the highest priorities to their GTA (Fig 2A; Table 3). While fewer students report that experiencing *Collaboration* (mean = 26.1% of each GTA’s students, SD = 12.9%) and *Iteration* (mean = 23.2%, SD = 12.1%) are among the top priorities to their GTA, the standard deviation for these statistics is still quite high (Fig 2B). This implies that students taught by some GTAs perceive that these elements are important to their GTA, while students of other GTAs feel these elements are not emphasized (Table 3):

*“Mostly everything in [the CURE] was overlooked*, *nothing was revised—it all seemed so unimportant and a waste of time and money.”—Student of GTA H, perceiving their GTA’s lack of emphasis on Iteration*

**Table 3:** Alignment of student and GTA perceptions of the importance of individual CURE constructs*.

CURE Construct	Students’ perceptions of construct importance to GTA	GTAs’ perceptions of construct presence in CURE
**Scientific Practices**	**Important to All GTAs** *“[Our GTA] has clearly demonstrated how to perform basic lab techniques*, *and the importance of why they need to be done correctly*. *They also stated that these techniques will be used in further research*.”–Student of GTA C, perceiving use of scientific practices	**Present in CURE** *“[Students don’t perceive using scientific practices]*, *I think*, *until the last four or five weeks because we start doing DNA extraction and gel electrophoresis and restriction enzyme digest*, *and those are the things that they learn about in lectures*. *And so*, *that’s what they associate with science… But I definitely think that they use scientific practices even in the beginning…”* –GTA C, perceiving use of scientific practices
**Collaboration**	**Important to Some GTAs** *“[Our GTA] was very clear that this lab was supposed to be collaborative*, *and we are supposed to gain knowledge from our classmates*.*”–*Student of GTA E, perceiving collaboration	**Present in CURE** *“They are working with partners and peers*. *[Teamwork is] very important*.* *.* *. *They are discussing with other students*, *sharing ideas*, *getting ideas*.*”*–GTA E, perceiving collaboration
**Iteration**	**Important to Some GTAs** *“[Our GTA] always says that the more we do it*, *the more we’ll understand*, *and the better we’ll get at it*.*”–*Student of GTA G, perceiving iteration	**Mostly Present in CURE** *“For most of the semester they’re just repeating the same thing to try to find phage*.* *.* *.*I allow students to revise their work once I give them their feedback to further improve their learning gains*. *Then I also push them all to think about how things are connected*. *I really try to hit iteration with my feedback*.*”–*GTA G, perceiving iteration
**Broader Relevance/ Novel Discovery**	**Not very important to GTAs** *“I highly doubt that the simple bacteriophage labs we do will create a huge influx in the science world*. *[Our GTA] does not [teach us] this”–Student of GTA D*, *perceiving lack of relevant discovery*	**Presence Varies by GTA** *“The bacterial hosts are not something that anyone cares about*.* *.* *. *if we were to do [the CURE] with a different host bacterium that could have actual medical relevance [students would experience relevant discovery]*.*”–*GTA D, perceiving lack of relevant discovery

*Quotes have been lightly edited for grammar, clarity, and to preserve anonymity of study participants.

In contrast to the variation seen for *Collaboration* and *Iteration*, few students report that *Novel Discovery* (mean = 13.7%, SD = 3.2%) and *Broader Relevance* (mean = 16.1%, SD = 2.5%) are highly prioritized by their GTAs—in fact, students more frequently reported that these items were least prioritized by their GTA, and this did not vary much by GTA (Fig 2A). In summary, students perceived that student engagement with *Scientific Practices* was important to their GTAs, *Collaboration* and *Iteration* were important to some of their GTAs, and *Broader Relevance* and *Novel Discovery* were not of particular importance to their GTAs (for supporting quotes, see Table 3).

We aligned these student perspectives of experiencing CURE elements with the perspective of their GTAs, which we explored through interviews. Most GTAs felt that students were exposed to multiple *Scientific Practices* and extensive *Collaboration* in the CURE (Table 3). GTA perceptions of *Iteration* in the CURE were slightly more variable—while most GTAs acknowledged that students experienced *Iteration* and some intentionally put extra effort into facilitating it (Table 3), GTAs D and J felt that iteration opportunities were limited:

*“[Students] really only repeat their work if something hasn’t worked…If they are successful*, *then they just keep moving on through these experiments, which I think is good because they get more excited about moving on and doing something new… I don’t know that iteration is necessarily something that we do a lot of in this course.”—GTA D*

GTAs varied the most in their perceptions that students experienced *Broader Relevance/Novel Discovery* through their participation in the CURE (Table 3). Five GTAs bought into the idea that students are experiencing this component of a CURE through their lab course, as students who successfully find a phage can contribute it to an online database:

*“The students know they are finding a novel phage… But the big impact on society is that they get to submit it to a database, which scientists can pull from. [The phages] can be involved in phage therapy*.*”–GTA C*

However, the remaining four GTAs (GTAs D, F, G, and H) felt this aspect of the course was limited, because they perceived that the scale of the potential *Novel Discovery* was very small, and/or the *Broader Relevance* to the greater scientific community was minimal:

*“But are you discovering something that’s going to be published? I think there is a deficiency with the SEA-PHAGES program and how it’s implemented*, *not just here but in other schools too, where the discovery might be limited. [Students] can put [their phage] into the database, but who knows if anyone’s going to look at it or use it in their own research that will lead to a publication.”–GTA G*

In summary, while both GTAs and students agreed that opportunities for students to experience multiple *Scientific Practices* were present in the course, students and GTAs did not always equivalently perceive the opportunities for the other CURE constructs. Though GTAs may perceive they are facilitating *Collaboration*, *Iteration*, and (sometimes) *Broader Relevance* and *Novel Discovery* in their courses, their students may not agree that these elements of research are emphasized.

### GTAs influence student beliefs regarding the purpose of participating in the CURE

To address our third research question, we asked students in an online survey to respond to the reflection question: “Why do you think your institution wants students to participate in the CURE?” In reviewing student responses to this question, we observed two distinct trends in students’ perceptions of the purpose of the CURE. Most students (78%) believed that their university engages students in CUREs for student-centered reasons (i.e., providing research experiences, helping develop lab skills and comfort in a lab setting, providing career/professional development, or increasing engagement with the course material). One student explained:

*“I believe that our institution wants students to participate in research-based curriculum in Biology lab because it is much more interactive and intuitive than a normal lab*. *We are actually conducting research and learning the processes of research and doing it on our own.”*

In contrast, 11% of students believed that their university employs CUREs in introductory biology labs solely for non-student-centered reasons (i.e., using students as a “free labor” resource to conduct research, using students to specifically further bacteriophage research, or because the CURE could bring more students or grant money to the institution):

*“[Our institution uses CUREs] to make the school look better*. *It is a top tier research school and unfortunately that aspect is taking over a plethora of courses. Our participation allows for more data collectors."*

An additional 11% of students expressed both beliefs, acknowledging that while the CURE lab may exist to advance the university or scientific research, it also serves to benefit the students who participate in the CURE.

**Fig 4:**
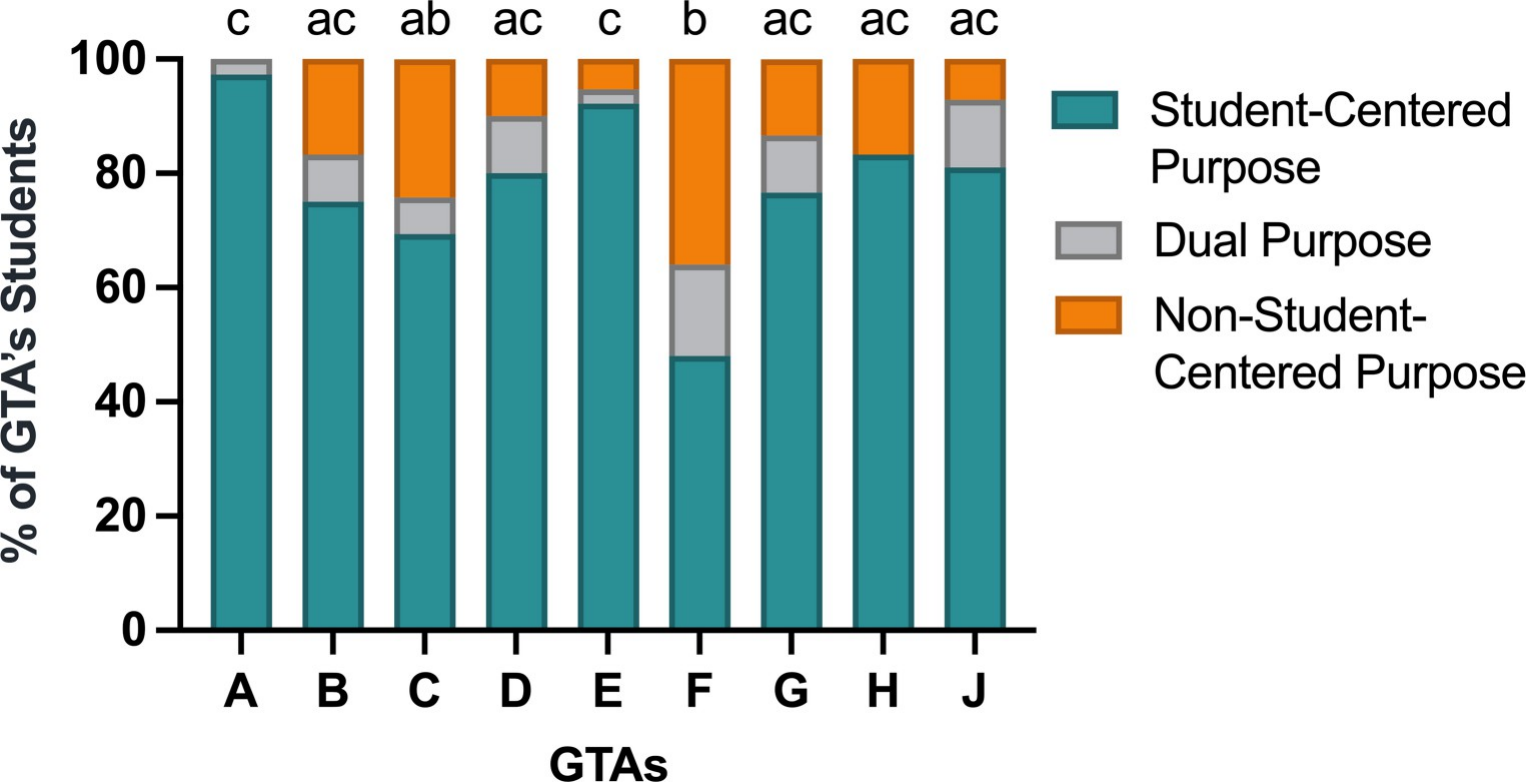
GTAs impact how students perceive the purpose of participating in a CURE. We coded student reflection questions to identify the proportion of each GTA’s students who believed that the university employed CUREs in introductory biology labs for student-centered purposes or non-student-centered purposes. Letters above bars indicate significant differences in the proportion of a GTA’s students who believe that the CURE has a non-student centered purpose: bars that do not share a common letter indicate a significant difference in perceived purpose for students of the indicated GTA. Most students believed that the CURE served a student-centered purpose; however, students of GTAs C and F more frequently reported that the CURE served a non-student centered purpose.

Kruskal-Wallis tests revealed that there were differences in the proportion of each GTA’s students who believed the purpose of the CURE was student-centered or not student-centered (Fig 4). Specifically, GTAs A and E had significantly higher proportions of students who believed the purpose of the CURE was student-centered as compared to GTAs C and F.

## Discussion

Here we found that student experiences in what are intended to be the ‘same’ CURE differ depending on the instructor. Students taught by different GTAs perceive notable differences in what is happening in the CURE classroom, the degree to which the CURE constructs are emphasized in their classes, and the purpose of participating in a CURE in introductory biology. Further, student perceptions of experiencing specific research elements in the CURE do not always align with GTA’s perceptions of facilitating those elements.

### Students who perceive a supportive learning environment experience more collaboration, iteration, and relevant discovery

Students perceived differing capacities among their GTAs to create supportive, positive learning environments, with the result that students of some GTAs feel encouraged and supported, while students of other GTAs feel anxious and uncomfortable (Fig 1). It has long been documented that student perceptions of instructor “misbehaviors,” such as the behaviors we coded as “Insufficient” and “Help!” in our focus group analyses, detract from students’ perceived experiences in a course [53]. Perceptions of instructor misbehaviors has also been hypothesized to contribute to student resistance to evidence-based learning pedagogy [54], and is linked to decreased student motivation to engage in a CURE [22].

We observed that students of GTAs who spoke the most about their GTA’s capacity to create a positive learning environment (such as GTAs A, B, and C; Fig 1), also reported the highest perceptions of engaging in *Collaboration*, *Iteration*, and *Broader Relevance/Novel Discovery* (Fig 3). Students who indicated that their GTAs did not create supportive lab environments (such as GTAs G, H, and J; Fig 1) scored lowest in their perceptions of the same critical CURE elements (Fig 3). These findings align with the results of many studies that have found that students who perceive that their instructors engage in supportive behaviors are more likely to experience positive affective and cognitive outcomes [22, 54–57], while unsupportive and antagonistic instructors can increase student anxiety and stress [58, 59], which can lead to decreased affect and cognitive learning for students [60].

Variation in the supportive environment created by individual GTAs likely therefore results in variation in experiencing elements of research and receiving the affective and cognitive benefits of participating in a CURE, as seen in other studies [22, 55, 56]. This potentially also perpetuates inequitable experiences of undergraduate research: positive perceptions of the lab environment is linked to increased persistence for students participating in apprentice-based undergraduate research experiences, and students who experience a negative lab environment are more likely to leave their research experiences [61]. As for students in apprentice-based research experiences, it is probable that perceptions of the research mentor and lab environment in the CURE would impact students’ interest in pursuing research experiences.

### GTAs emphasize different learning priorities and communicate different purposes of the CURE

In addition to perceiving differences in the lab environment as shaped by their GTA, students perceive that their GTAs vary in how they prioritize various learning objectives (Fig 2). It is a logical assumption that individual instructors will vary in their behaviors and communication styles. Variation in faculty and GTA instructor behavior has been documented in analyses of introductory biology classrooms using the Instructor Talk framework, which allows for systematic documentation and analysis of the non-content communication instructors relate to students, such as talk that builds interpersonal relationships, establishes classroom culture, explains pedagogical choices, and negatively phrased talk [62–64]. Fewer studies focus on variation in GTA behavior when teaching lab sections of the same course [27, 29, 65]. Our own interview analysis with the GTAs participating in this study indicates that GTAs teaching the same CURE can have vastly different conceptions of their instructor role, which likely influences the instructional decisions made by GTAs [27]. We expect that some of the variation that students perceive about what is most or least prioritized in the classroom (Fig 2B, Fig 2C) is a direct reflection of the effort GTAs put toward providing students with those experiences. The high variation we observed in student perceptions of GTA classroom priorities such as collaboration, iteration, and independent troubleshooting indicate student experiences with these elements could be appreciably different, depending on their GTA.

We hypothesize that differences in GTA instructor talk [62–64] and variation in messaging from GTAs may contribute to differences in students’ overall understanding of why they are participating in research-based curriculum (Fig 4). Recent work has found that GTAs engage in negatively-phrased instructor talk, defined as talking in a way that discourages students or distracts from learning, at higher rates than faculty instructors[64]. All GTAs in our study participated in a training ‘boot camp’ where they were presented papers and data that support the pedagogical advanatages of engaging students in a CURE; however, the degree to which GTAs communicated this information to their students likely varied. GTAs who explained the pedagogical rationale for implementing a CURE, as well as the potential benefits to students from participating in a CURE, may have contributed to students’ beliefs that they were participating in the CURE for student-centered purposes. In interviews conducted with the nine GTAs involved in our study (presented in [27]), we observed that a couple of the GTAs doubted the value for students of participating in a CURE, particularly at an introductory level. While documentation of instructor talk was beyond the scope of this study, it is possible that these GTAs may have actively engaged in unproductive instructor talk, such as expressing their doubt about the pedagogical advantages of the curriculum to their students [62]. This verbage could contribute to student beliefs that they were participating in a CURE for non-student-centered purposes, which were often expressed with a adverse tone (i.e., to benefit the university or use students as “free labor” in advancing research projects). Instructors who do not sufficiently convey the pedagogical advantages of the CURE may contribute to decreased student buy-in and increased student resistance to engaging in the CURE [63].

### Students and GTAs believe that scientific practices, collaboration, and iteration take place in the CURE

Though there was some variation in student perceptions of how important these elements were to their individual GTAs, students overall identified that their GTAs highly prioritized the CURE research constructs of *Scientific Practices*, *Collaboration*, and *Iteration* (Fig 2, Table 3). GTAs generally agreed that these elements were prioritized in their classes (Table 3). Given that the intention of a CURE is to replace content-delivery style laboratory experiences with opportunities for students to experience the process of science, we were surprised that students perceived the top GTA priorities were “Scientific Practices” and “Understanding the bacteriophage system.” Previous studies have shown that students rarely have a clear understanding of the purpose of their participation in traditional scientific laboratory settings, and the ones that do generally perceive that the purpose is simply to “follow instructions” or “get the right answer” [66]. The emphasis students placed on the importance of experiencing “Scientific Practices” and “Understanding the bacteriophage system” could be interpreted similarly to the broader objectives of “following instructions” or “getting the right answer,” because students often experienced scientific practices through following the instructions of their GTA or lab protocols. Additionally, students were given GTA-led lectures and quizzes on both their research methods and the bacteriophage-host context of their project, which likely reinforced the perceived importance of these elements within the CURE curriculum.

Though students reported experiencing *Iteration*, they also largely felt that “independent troubleshooting” was unimportant to their GTA (Fig 2). While “independent troubleshooting” on its own is not one of the five elements defined in the CURE, there is an expectation that students engage in this activity as part of the experience of *Iteration*: as stated by Auchincloss and colleagues (2014), “students learn by trying, failing, and trying again.” It was therefore striking that so many students said that “independent troubleshooting” was one of the lowest priorities for their GTAs (Fig 2A). This may be an indication that CURE GTAs, like inquiry GTAs, have difficulty in providing space for students to learn through struggle [67]. Recent work has found that experiences of “failure” and challenges in a CURE can be beneficial, and can increase student buy-in, resiliency in navigating obstacles, and understanding of the nature of science [20, 68, 69]. Yet, these studies on the benefits of failure and facing challenges during a CURE all highlighted the importance of psychosocial support from the instructor in order to help students understand and learn from these experiences[20, 68, 69]. Further, instructor actions and dispositions are thought to be a key part to supporting students to develop adaptive coping behaviors in response to the challenges and failures they encounter in their learning environments[70]. Therefore, GTAs who limit opportunities for students to learn through failure, or who fail to provide appropriate psychosocial support to allow students to productively cope and learn through failure, may reduce the potential benefits of participating in a CURE.

### GTAs and students are uncertain of the broader relevance and novel discovery in the SEA-PHAGES curriculum

The CURE constructs of *Broader Relevance* and *Novel Discovery* distinguish a CURE from other types of laboratory curriculum by scaffolding the opportunity for students to engage in authentic research [13, 20, 21]. However, few students felt that the *Broader Relevance* or *Novel Discovery* elements of research were emphasized by their GTAs (Fig 2, Table 3). The fact that students did not see these elements as important could be a consequence of any combination of phenomena, including: 1) students are not recognizing the presence or importance of *Broader Relevance* and *Novel Discovery*; 2) GTAs do not emphasize or effectively scaffold the elements of *Broader Relevance* and *Novel Discovery* in their classes; and/or 3) the elements of *Broader Relevance* and *Novel Discovery* are not sufficiently scaffolded within the design of the SEA-PHAGEs curriculum itself.

It is likely that all three of the phenomena posed above are at play in limiting students’ perceptions of *Broader Relevance* and *Novel Discovery*. Previous studies have found that students are unable to identify the skills, concepts, and teaching practices used in their courses [66, 71, 72]. In addition to having limited perceptions of the purpose of their laboratory courses [66], students and instructor perceptions of the instructional practices used in a course are not strongly correlated [71]. Further, students can be unaware of specific concepts that were taught in their class, even when instructors and expert observers report that those concepts were addressed [72]. It therefore is plausible that students do not recognize the presence of *Broader Relevance* and *Novel Discovery* when these constructs have been scaffolded into the CURE.

GTAs, too, often doubted the presence of *Broader Relevance* and *Novel Discovery* in the CURE. SEA-PHAGES curriculum scaffolds these elements such that students isolate a phage, which is presumed to be novel due to the wide diversity of bacteriophages (*Novel Discovery*), and archive information about the phage in an online national database, where the information could potentially be used by other scientists (*Broader Relevance*). While all GTAs were aware of the novelty of the isolated phage and the contribution of information to the online database, nearly half of the GTAs still said that these elements are insignificant in the curriculum (Table 3). Therefore, it is likely that some GTAs had difficulty communicating the *Broader Relevance* and *Novel Discovery* of the research project to their students because GTAs felt that these elements were not sufficiently developed in the curriculum itself. It is also possible that GTAs’ perceptions of *Broader Relevance* and *Novel Discovery* is influenced by their own understanding of what it means to do science—for example, if their own projects and research interests are centered around hypothesis-driven research, they may recognize less relevance in the discovery-driven SEA-PHAGES curriculum. Further research could explore GTA conceptions of science and how this impacts their ability to teach CUREs and inquiry-based labs.

### Are the elements of broader relevance and novel discovery sufficiently scaffolded within the SEA-PHAGES curriculum?

The SEA-PHAGES curriculum is an example of a network CURE. Unlike an independent CURE, where students conduct research that contributes to a faculty member’s research program or the local community, a network CURE is developed and packaged such that it can be taught on a large scale by faculty members at multiple institutions [73]. The failure of students to recognize the elements of *Broader Relevance* and *Novel Discovery* is inconsistent with the experiences of students of independent CUREs, who both recognize *Broader Relevance* and *Novel Discovery* and experience positive affective outcomes associated with these elements [20, 21]. Since, in our study, we found that both students and instructors perceive that these elements are lacking, we question to what extent students who participate in the SEA-PHAGES curriculum are actually experiencing the research experience that is intended with the CURE model [13].

Implementation of the SEA-PHAGES curriculum varies widely, and students who participate at other institutions may have the opportunity to participate in activities not available to the students in our study. These activities include sequencing their phage genome, spending a second term of research conducting bioinformatic analyses on their isolated phage, and/or participation in local or national meetings [34]. Many institutions do not have the capacity to provide all of these elements of the SEA-PHAGES curriculum to students, and there is evidence of positive student outcomes from participating in just the phage discovery portion of the course (as students in our study experienced) [37]. Though the SEA-PHAGES curriculum provides many academic and affective benefits for students [34, 36, 37], we are unaware of other studies that measure SEA-PHAGE student perceptions of experiencing the CURE constructs, including *Broader Relevance* and *Novel Discovery*. Future research could explore whether *Broader Relevance* and *Novel Discovery* are adequately scaffolded in network CURE curricula such as to meet the expectations of a CURE. It is possible that students participating in the SEA-PHAGES curriculum are more closely experiencing an advanced inquiry-style course—which can still benefit students and allow for students to experience elements of authentic research [20, 21]—rather than a true CURE.

### How do individual GTAs impact their students in a CURE?

Cumulatively, our data indicate that students taught by different GTAs can experience different learning environments (Fig 1); perceive that their instructors have different priorities in the classroom (Fig 2); experience elements of the CURE differently (Fig 3); and have different perceptions of the purpose of participating in a CURE (Fig 4). While our evidence supports that different GTAs foster different experiences for their students, identifying GTA-level characteristics that result in these different experiences will be challenging. However, we can make some observations about patterns in our findings that could be explored with further studies between CURE GTAs and their students.

We found that students of GTAs A, B, and C most frequently discussed the supportive environment their GTA created (Fig 1). These students also reported experiencing the highest level of research elements in their class (Fig 3), indicating that their GTAs were likely effective in providing structural and psycosocial support for their students while supporting their student’s awareness in experiencing aspects of research in the CURE. Interestingly, students of these GTAs did not all agree on the purpose of participating in a CURE—students of GTA A reported a highly student-centered purpose, while greater proportions of students taught by GTAs B and C perceived non-student-centered purposes. Notably, from the GTA perspective, GTAs A, B, and C all described that the CURE included sufficient opportunities for *Iteration* and *Novel Discovery/Broader Relevance*—in contrast to several of the other GTAs. This indicates some alignment between a GTA’s beliefs that these research opportunities are present in the CURE curriculum, and their students’ perceptions of experiencing research elements in a CURE (Table 3, Fig 3). Interstingly, LCAS scores indicate that GTAs A, B, and C perceive facilitating these research elements at comparable levels as their students perceive experiencing these research elements, while other GTAs significantly overestimated the degree to which they perceived facilitating research elements compared to their students’ perceptions (Fig 3). High GTA LCAS scores, then, may not be an accurate predictor for the degree to which students will perceive experiencing research elements in their CURE.

Our study included nine GTAs who came in to their CURE teaching role with varying amounts of teaching experience, interest in a teaching career, and experience as a researcher. All of these factors, as well as a GTA’s discipline of study, gender, culture and nationality, and other intersectional demographic variables could impact a GTA’s willingness to buy-in to teaching the CURE curriculum, as well as their competence in doing so. Given the myriad of potential variables and our small sample size, we are unable to provide evidence regarding the impact that these specific variables might have had on GTA instruction. However, holistically, we did not observe apparent connections between a GTA’s students’ outcomes and the GTA’s teaching experience, interest in a teaching career, or discipline of study. Additionally, while we did not observe any connections between individual GTAs and their students’ success in isolating and purifying their own novel phage, interviews with students who participated in the SEA-PHAGES CURE at our study institution revealed that success in discovering a novel phage did positively impact student’s intrinsic motivation in the course [22]. A GTA’s ability to accurately guide students through the experiments, and to provide the psychosocial support to encourage students to persist and engage in opportunities for iteration, could therefore impact student outcomes in a CURE. Further research should explore how all of these GTA experiences and characteristics might impact a GTA’s instructional behaviors and student outcomes in a CURE.

### Limitations

The findings from this study represent the experiences and perspectives from a single set of GTAs and their students during one term of an introductory biology lab course at one institution. It is likely that the experiences and perceptions of students and instructors would be different given other contexts, such as course level (upper vs lower division), institution, GTA training, selection of GTA instructors, and variation in CURE curriculum. Despite this limitation, our work demonstrates the potential range and variation of experiences that students may have when taught by different GTAs in a single CURE. We therefore encourage practitioners and researchers to be cognizant of the types of impacts that individual GTAs may have on their students’ experiences in a CURE.

Despite evidence that the LCAS survey functions well in similar populations at other institutions [20, 44, 74, 75], our data-model fit statistics indicate that there may be some issues with the LCAS item functioning for our student population at this institution (further discussed in S2 text). Though the LCAS was developed for use with undergraduates in research-based courses, our study corroborates others that have found issues with model-fit with their own student populations [20, 74]. We therefore strongly recommend that instructors and researchers who use the LCAS conduct confirmatory factor analyses to assess LCAS model fit, to determine if adjustments to the instrument need to be made in their own student population and to aid in appropriate interpretation of results. We decided to continue to include the LCAS data in this manuscript because the fit, while below recommended cutoffs, is still reasonable, and the trends we see in the LCAS data align with the trends seen in other data used in this manuscript.

## Conclusions and recommendations

Patterns of variation in perceptions of the lab environment and course intentions reveal that students of different GTAs may have fundamentally different experiences, even when they are engaged in the same course and curriculum. Some GTAs likely are facilitating learning environments more conducive to achieving the benefits intended for students by participating in a CURE, while other GTAs are not. Equity of student experiences in GTA-taught CUREs should therefore be a concern of future researchers. Are students still benefiting from a CURE when taught by a GTA (or any instructor) who creates an unsupportive learning environment, or fails to understand or emphasize critical CURE elements? What training and characteristics does a GTA need to be successful in teaching a CURE effectively? Additionally, as differences in instruction clearly exist in GTA-taught laboratory courses, researchers should also consider GTAs as a variable in future analyses exploring student experiences in laboratory classes.

Practitioners will want to provide professional development opportunities to better prepare GTAs to effectively facilitate research-based curricula for their students. Training should focus not only on the technical aspects of teaching scientific tools and processes, but also on creating supportive learning environments for students, facilitating effective student engagement with the critical elements outlined in the CURE framework, and using positive instructor talk to explain the purpose of participating in research-based curricula. Educators leading GTA-taught CUREs should look for opportunities to optimize instructor messaging on the purpose and potential benefits of a CURE experience through means additional to GTAs. For example, if there are associated lecture sections taught by a faculty member, the faculty member could engage all students in discussions of the CURE, or information about the purpose and benefits of a CURE could be integrated into videos or instructions that all students receive, regardless of their GTA.

Lastly, network CUREs could consider leveraging the structure and resources they have already developed to offer CURE-specific training for their GTAs, or training for faculty instructors on how to best support GTAs. For example, the HHMI SEA-PHAGES program already offers training workshops for new faculty instructors, but to our knowledge this training is not available to GTAs and does not address how faculty could offer CURE-specific GTA training [76].

By implementing CUREs at the introductory biology level, there is an implicit assumption that we are providing a structured, equitable research experience for students by increasing and standardizing opportunities for students to participate in research. However, depending on both the curriculum and the instruction, students may fail to sufficiently experience the critical research elements defined in the CURE framework. Researchers and educators should continue to consider the presence of *Broader Relevance* and *Novel Discovery* within SEA-PHAGES and other CURE curricula and consider ways to strengthen the intentionality of these constructs to provide a true research experience for students.

## Supporting information

S1 Table Student focus group codes and GTA competency categorization. (PDF)

S1 Text Student lab priorities task and items. (PDF)

S2 Text Laboratory course assessment survey information and analysis. (PDF)

S1 Dataset Student priorities worksheet responses. (XLSX)

S2 Dataset Laboratory course assessment survey student responses. (XLSX)
