# Supplementary material for: Not the same CURE: Student experiences in course-based undergraduate research experiences vary by graduate teaching assistant
Source: PLoS One. 2022 Sep 27;17(9):e0275313. doi: 10.1371/journal.pone.0275313 (PMC9514618; doi:10.1371/journal.pone.0275313)
Supplement: S1 Table — (PDF) [file pone.0275313.s001.pdf]

S1 Table. Student Focus Group Codes and GTA Competency Categorization

Theme	Code	GTA Competency Categorization
**GTA STRENGTHS**		
Students perceive GTA as a "good" instructor	Invested in students and teaching	Above and Beyond
	Used inclusive/effective teaching techniques	Above and Beyond
	Encourages student critical thinking	Above and Beyond
	Students really appreciate GTA	Above and Beyond
	GTA was reflexive/adaptive to student needs	Above and Beyond
	GTA fosters student investment/motivation in lab	Above and Beyond
	GTA had a very positive attitude	Baseline
	GTA was organized	Baseline
	GTA was flexible	Baseline
GTA is a strong communicator	Communicates expectations well	Baseline
	Communicates at appropriate level for students	Baseline
	Explained experimental procedures well	Baseline
	Explained experimental purpose well	Baseline
	Most students understand purpose of CURE research project	Baseline
	Thorough feedback on reports/final paper/lab progress	Baseline
	Responds to emails promptly	Baseline
GTA creates a positive lab environment	GTA creates comfortable environment	Baseline
	GTA creates productive environment	Baseline
	GTA creates collaborative environment	Baseline
**GTA WEAKNESSES**		
GTA is a poor communicator	Clearer expectations, and consistency needed in course	Insufficient

	Needed more instructional clarity or guidance for lab procedures	Insufficient
	Need more/higher-quality feedback on assignments	Insufficient
	Instructor was confused about lab activities/quizzes	Insufficient
	Instructor unnecessarily over-explained things	Insufficient
	Students need more purpose for each individual experiment	Insufficient
	Communicates at higher level than student understanding	Help!
	Most students don’t understand purpose of CURE research project	Help!
	Most students don’t understand overall purpose of participating in research-based lab	Help!
Students perceive GTA as a "bad" instructor	More organization needed from GTA	Insufficient
	GTA did not effectively use class time	Insufficient
	Lack of engagement from GTA	Help!
	Need more encouragement from GTA	Help!
	GTA creates stressful environment	Help!
	GTA has unreasonably high standards	Help!
	More support needed from GTA	Help!
