# Supplementary material for: Not the same CURE: Student experiences in course-based undergraduate research experiences vary by graduate teaching assistant
Source: PLoS One. 2022 Sep 27;17(9):e0275313. doi: 10.1371/journal.pone.0275313 (PMC9514618; doi:10.1371/journal.pone.0275313)
Supplement: S1 Text — (PDF) [file pone.0275313.s002.pdf]

S1 Text. Student Lab Priorities Task and Items

<u>Student Instructions for Lab Priorities Task:</u>

Below is a list of potential Biology 107 laboratory ***learning objectives an instructor may have for their students***. Consider each objective, and mark **three objectives** you believe may be the most important and **three objectives** you believe may be the least important <u>*to your lab instructor.*</u>

Pick **one** of the objectives that you listed as **most important.** Please provide a specific example of how your lab instructor has indicated (verbally or through their actions) that this is a priority for them.

Pick **one** of the objectives that you listed as **least important.** Please provide a specific example or explanation for why you believe this is not a priority for your lab instructor.

**Full Lab Priority Item**	**Shortened Name**
Students learn how to analyze and interpret data.	Data analysis/interpretation
Students collaborate with teammates to work on a scientific project.	Collaboration
Students better understand the content of the associated lecture course.	Lecture reinforcement
Students learn if research is a career they would like to pursue.	Career clarification
Students become excited about research and science.	Excitement for research
Students conduct an investigation to discover something previously unknown to the scientific community.	Discovery
Students produce accurate and reliable scientific data.	Produce accurate data
Students enjoy their time in the CURE lab.	Enjoy lab
Students learn the importance of revising or repeating their work to improve the quality of their research.	Values iteration
Students learn to troubleshoot problems independently.	Independent troubleshooting
Students develop basic lab skills (learn how to pipette, do a plaque assay, etc.).	Scientific practices
Students feel comfortable asking their instructors questions or discussing any problems.	Approachable instructor
Students develop an understanding of bacteriophages and host system.	Understanding bacteriophage system
Students learn the process of conducting research.	Process of research
Students work on a research project that has the potential to make a real contribution to the public or the scientific community.	Broader relevance
