# Supplementary material for: Not the same CURE: Student experiences in course-based undergraduate research experiences vary by graduate teaching assistant
Source: PLoS One. 2022 Sep 27;17(9):e0275313. doi: 10.1371/journal.pone.0275313 (PMC9514618; doi:10.1371/journal.pone.0275313)
Supplement: S2 Text — (PDF) [file pone.0275313.s003.pdf]

S2 Text. Laboratory Course Assessment Survey Information and Analysis

Table A. Items and Response Options*

Construct	Prompt	Item	Item Text	Item Response Options
Collaboration	I was encouraged to…	C1	discuss elements of my investigation with classmates or instructors	1: Never<br>2: Only once<br>3: A couple of times, but not every lab period<br>4: About once per lab period<br>5: Multiple times during most lab periods
		C2	reflect on what I was learning	
		C3	contribute my ideas and suggestions during class discussions	
		C4	help other students collect or analyze data	
		C5	provide constructive criticism to classmates and challenge each other's interpretations	
		C6	share the problems I encountered during my investigation and seek input on how to address them	
Broader Relevance/ Novel Discovery	I was expected to…	D1	generate novel results that are unknown to the instructor and that could be of interest to the broader scientific community or others outside of class	1: Strongly disagree<br>2: Disagree<br>3: Somewhat disagree<br>4: Somewhat agree<br>5: Agree<br>6: Strongly agree
		D2	conduct an investigation to find something previously unknown to myself, other students, and the instructor	
		D3	formulate my own research questions or hypothesis to guide an investigation	
		D4	develop new arguments based on data	
		D5	explain how my work has resulted in new scientific knowledge	
Iteration		I1	revise or repeat work to account for errors or fix problems	
	I had time to…	I2	change the methods of the investigation if it was not unfolding as predicted	
		I3	share and compare data with other students	
		I4	collect and analyze additional data to address new questions or further test hypotheses that arose during the investigation	
		I5	revise or repeat analyses based on feedback	
		I6	revise drafts of papers or presentations about my investigation based on feedback	

** Adapted from Corwin et al. (2015). Note that original item response options for the* Collaboration *scale are as follows: 1= Never; 2= One or two times; 3= Monthly, 4= Weekly.*

Table B. LCAS Item Summary Statistics*

Items	Mean	SD	Median	Min	Max	Skewness	Kurtosis
Collaboration							
C1	3.799	1.068	4	1	5	-0.821	3.251
C2	3.893	0.961	4	1	5	-0.956	3.999
C3	3.530	1.125	4	1	5	-0.495	2.779
C4	3.460	1.214	3	1	5	-0.503	2.557
C5	3.201	1.146	3	1	5	-0.346	2.617
C6	3.619	1.076	4	1	5	-0.65	3.064
Broader Relevance/ Novel Discovery							
D1	4.133	1.256	4	1	6	-0.602	2.884
D2	4.332	1.242	4	1	6	-0.836	3.503
D3	3.883	1.33	4	1	6	-0.366	2.479
D4	3.906	1.256	4	1	6	-0.457	2.797
D5	4.355	1.173	4	1	6	-0.823	3.712
Iteration							
I1	4.692	1.123	5	1	6	-0.862	3.649
I2	4.026	1.312	4	1	6	-0.565	2.694
I3	4.198	1.271	4	1	6	-0.767	3.106
I3	4.198	1.271	4	1	6	-0.767	3.106
I4	4.084	1.31	4	1	6	-0.652	2.923
I5	4.407	1.222	5	1	6	-0.952	3.681
I6	4.640	1.228	5	1	6	-1.215	4.374
I3	4.198	1.271	4	1	6	-0.767	3.106

*Summary statistics for items in each of the three LCAS scales (Collaboration, Discovery, and Iteration). Our items show little skew (all absolute skewness values are less than 1.5), and moderate kurtosis (ranging between 2.4 and 4.4). Acceptable absolute kurtosis values for normal data range from below 2.0 ("conservative", Hancock et al., 2018) to below 7.0 ("liberal", Hancock et al., 2018) or even below 10.0 ("conservative"; Kline, 2015). To account for this moderate non-normality of our data, we used a robust estimator in our confirmatory factor analysis.

LCAS Reliability and Data-Model Fit

We use the omega reliability coefficient, rather than Cronbach's alpha, as an estimate of the internal consistency of our three instrument scales, as the omega reliability coefficient is equivalent to Cronbach's alpha when factor loadings are equivalent and avoids bias introduced by Cronbach's alpha when factor loadings are independent (Komperda, Hosbein, et al., 2018; Komperda, Pentecost, et al., 2018). McDonald's Omega total for the Collaboration, Iteration, and Discovery/Relevance subscales was 0.86, 0.88, and 0.88 respectively, indicating that all three subscales have acceptable internal consistency (reliability coefficients above 0.8 are considered "very good"; Kline, 2015).

While historical recommendations for cut-off values for incremental fit indices such as the comparative fit index (CFI) and the Tucker-Lewis Index (TLI) have suggested that models with values above 0.90 may be acceptable, current sources recommend that values for these indices should be 0.950 or above to indicate good model fit (Hancock et al., 2018; Hu & Bentler, 1999; Schermelleh-Engel et al., 2003). The root mean square error of approximation (RMSEA) is a parsimony-adjusted badness-of-fit index, and guidelines recommend values for the RMSEA should fall at or below 0.08 for "acceptable" model fit and at or below 0.05 for "good" model fit (Hu & Bentler, 1999; Kline, 2015; Schermelleh-Engel et al., 2003).

As seen in the table below, fit indices for our tested three-factor model suggest that though model fit is not terrible, it is at or below recommendations for "acceptable" model fit. We therefore have chosen to not use the LCAS survey data as evidence for claims central to this study, but rather as supporting evidence for other data within this study that show similar trends in the perceptions of students taught by individual GTAs.

Table C. LCAS Data-Model Fit

**Fit Indices**	**Data-Model Fit**	**Acceptable Fit Guidelines***
CFI	0.922	≥ 0.950
TLI	0.909	≥ 0.950
RMSEA (90% confidence)	0.080 (0.069-0.091)	≤ 0.080

*As suggested by Schermelleh-Engel et al. (2003) and Hancock et al. (2018)

References

Corwin, L. A., Runyon, C., Robinson, A., & Dolan, E. L. (2015). The laboratory course assessment survey: A tool to measure three dimensions of research-course design. *CBE—Life Sciences Education*, *14*(4), ar37.

Hancock, G. R., Stapleton, L. M., Mueller, R. O., Stapleton, L. M., & Mueller, R. O. (2018). *The Reviewer's Guide to Quantitative Methods in the Social Sciences*. Routledge. https://doi.org/10.4324/9781315755649

Hu, L., & Bentler, P. M. (1999). Cutoff criteria for fit indexes in covariance structure analysis: Conventional criteria versus new alternatives. *Structural Equation Modeling: A Multidisciplinary Journal*, *6*(1), 1–55. https://doi.org/10.1080/10705519909540118

Kline, R. B. (2015). *Principles and Practice of Structural Equation Modeling, Fourth Edition*. Guilford Publications.

Komperda, R., N. Hosbein, K., & Barbera, J. (2018). Evaluation of the influence of wording changes and course type on motivation instrument functioning in chemistry. *Chemistry Education Research and Practice*, *19*(1), 184–198. https://doi.org/10.1039/C7RP00181A

Komperda, R., Pentecost, T. C., & Barbera, J. (2018). Moving beyond Alpha: A Primer on Alternative Sources of Single-Administration Reliability Evidence for Quantitative Chemistry Education Research. *Journal of Chemical Education*, *95*(9), 1477–1491. https://doi.org/10.1021/acs.jchemed.8b00220

Schermelleh-Engel, K., Moosbrugger, H., & Müller, H. (2003). *Evaluating the Fit of Structural Equation Models: Tests of Significance and Descriptive Goodness-of-Fit Measures*. *8*(2), 52.
